# GM2 gangliosidosis AB variant: novel mutation from India – a case report with a review

**DOI:** 10.1186/s12887-016-0626-6

**Published:** 2016-07-11

**Authors:** Jayesh Sheth, Chaitanya Datar, Mehul Mistri, Riddhi Bhavsar, Frenny Sheth, Krati Shah

**Affiliations:** Department of Biochemical and Molecular Genetics, FRIGE’s Institute of Human Genetics, FRIGE House, Jodhpur Gam Road, Satellite, Ahmedabad, 380015 Gujarat India; Sahyadari Medical Genetics and Tissue engineering facility (SMGTEF), Pune, 411005 India

**Keywords:** GM2 gangliosidosis, *GM2A* gene, GM2 activator protein, AB variant

## Abstract

**Background:**

GM2 gangliosidosis-AB variants a rare autosomal recessive neurodegenerative disorder occurring due to deficiency of GM2 activator protein resulting from the mutation in *GM2A* gene. Only seven mutations in nine cases have been reported from different population except India.

**Case presentation:**

Present case is a one year old male born to 3rd degree consanguineous Indian parents from Maharashtra. He was presented with global developmental delay, hypotonia and sensitive to hyperacusis. Horizontal nystagmus and cherry red spot was detected during ophthalmic examination. MRI of brain revealed putaminal hyperintensity and thalamic hypointensity with some unmyelinated white matter in T2/T1 weighted images. Initially he was suspected having Tay-Sachs disease and finally diagnosed as GM2 gangliosidosis, AB variant due to truncated protein caused by nonsense mutation c.472 G > T (p.E158X) in *GM2A*gene.

**Conclusion:**

Children with phenotypic presentation as GM2 gangliosidosis (Tay-Sachs or Sandhoff disease) and normal enzyme activity of β-hexosaminidase-A and -B in leucocytes need to be investigated for GM2 activator protein deficiency.

## Background

GM2 gangliosides are the glycosphingolipids present in the outer layer of mammalian cells that are enriched on the neuronic surfaces [1]. In normal condition, glycosphingolipids are catabolized by lysosomal exohydrolases and are unique as they require synthesis and interaction of three-gene products;α and β subunits of lysosomal glycosidase enzyme-β-hexosaminidase (EC 3.2.1.52) and presence of a small non-enzymatic lipid binding protein as an activator [1]. This tiny glycolipid transporter GM2 activator protein (GM2AP), acts as a substrate specific cofactor for the degradation of GM2 ganglioside by the enzyme β-hexosaminidase. Hence, deficiency of any of these protein that are encoded by the *HEXA*, *HEXB* and *GM2A* gene causes excessive intra lysosomal accumulation of GM2 and related glycolipids especially in neuronal cells resulting in GM2 gangliodosis [2].

Mutations in the *HEXA* gene encoding the α-chain of (β-hexosaminidase-A) Hex-A leads to Tay-Sachs disease or B variant, while mutations in the β-chain encompassing *HEXB* gene leads to deficiency of both Hex-A and Hex-B (total-Hex) causing Sandhoff disease or O variant. These disorders are fairly common in the Indian population [3]-[4]. The third variant of GM2 gangliosidosis, known as AB variant (OMIM-272750), is rarely encountered and only nine cases are reported till date world wide as described in Table 1 [2], [5]-[6]. This form of GM2 gangliosidosis is indistinguishable from infantile form of Tay-Sachs disease due to its phenotypic similarity. Here we report a novel case with a review of GM2A activator protein deficiency.

**Table 1:** Review of molecularly proven cases of GM2 Gangliosidosis-AB variant

Case	Mutation	Exon	Predicted protien change	Author	Ethnicity	Year
1	c.412T>C (p.C107R)^a^ (Homozygous)	3	Reduced interaction with Hex A	Schroder et al. [15]	US Black	1991
2	c.412T>C (p.C138R)^a^ (Homozygous)	3	Reduced interaction with Hex A	Xie et al. [16]	US Black	1992
3	c.506G>C (p.R169P) (Homozygous)	4	Pre-matured protein degradation	Schroder et al. [17]	Indian	1993
4	c.262_264delAAG(p.88Kdel) (Homozygous)	3	Absence of mature CRM	Schepers et al. [14]	Saudi Arabia	1996
5	c.410delA (p.H137PfsX34) (Homozygous)	3	Absence of mature CRM	Schepers et al. [14]	Spanish	1996
6	c.160G>T (p.E54X) (Homozygous)	2	Absence of mRNA or CRM	Chen et al. [9]	Laotian, Hmong	1999
7	c.522T>G (p.L174R) (Homozygous)	4	Pre-matured protein degradation	Kolodny et al. [18]	Indian	2008
8	c. 160G>T (p.E54X) (Homozygous)	2	Absence of mRNA or CRM	Renaud et al. [19]	Hmong	2015
9	c.164C>T (p.P55L) (Homozygous)	2	Reduced interaction with Hex A	Salih et al. [6]	Saudi Arabia	2015
10	c.472G>T (p.E158X) (Homozygous)	4	Absence of mRNA or CRM	Present case	Indian	2015

^a^ The mutations identified by Schroder et al. (1991) (CYS107ARG) and Xie et al. (1992) (CYS138ARG) are the same but derived from different amino acid numbering systems

## Clinical presentation

Proband was a third child born to young third degree consanguineous parents from Maharashtra. One elder sister died at the age of 1½ years having similar complaints.

The case under report had a normal antenatal and perinatal history and was born with a birth weight of 3.05 kg at term. At the age of 12 months, global development delay with absence of social smile and poor head control was noticed. He was detected having hypotonia with a significantly poor tone in the limbs compared to the axial movements. The visual responses seemed quite poor. Head circumference was normal and no dysmorphism was observed. The child did not show any Mongolian patches on lumbosacral area which was detected, in our earlier case series with Tay-Sachs disease [7].

**Fig. 1:**
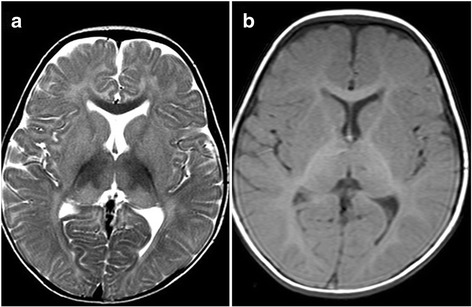
**(a)-(b)**: Initial T2 weighted MRI pictures of brain revealed (**a**) putaminal hyperintensity and (**b**) thalamic hypointensity with some unmyelinated white matter in T2/T1 weighted images

On clinical examination, there was no organomegaly. Significant hyperacusis was noted even to the most trivial sounds. Fundus examination showed bilateral cherry red spot in macula and horizontal nystagmus. Magnetic Resonance Imaging (MRI) scan of brain revealed putaminal hyperintensity and thalamic hypointensity with some unmyelinated white matter in T2/T1 weighted images (Fig 1(a)-(b)), which is commonly detected in cases with GM2 gangliosidosis.

After obtaining institutional ethics committee approval and informed written consent, further study was carried out. The blood samples of the proband and their parents were taken for the study. Clinical presentation was pointing towards GM2 gangliosidosis. Enzyme activity was determined by fluorimetric method using specific synthetic substrate. Hex-A was assayed with a sulphated substrate 4-methylumbelliferyl-N-acetyl-β-D-glucosamine-6-sulphate (MUGS) whereas total hexosaminidase (total-Hex) was measured from the hydrolysis of the synthetic substrate 4-methylumbelliferyl-Nacetyl-β-D-glucosamine (MUG) that releases fluorescent 4-methylumbelliferone when acted upon by β-hexosaminidase [8]. Enzyme activities for Hex-A [102 nmol/hr/mg protein; NR: 69.0 - 659.0 nmol/hr/mg protein] and total-Hex [414.9 nmol/hr/mg protein; NR: 288.4 – 1758.0 nmol/hr/mg protein] were found to be normal. This makes it highly unlikely for Tay-Sachs or B variant and Sandhoff disease or O variant. In presence of strong clinical presentation and normal lysosomal enzyme activity; GM2 gangliosidosis activator deficiency leading to AB variant was carried out by mutation analysis encompassing GM2 activator (*GM2A*) gene.

Molecular analysis was carried out by bi-directional sequencing of the coding region of *GM2A* gene together with intronic flanking region using the primer pairs as described earlier [9]. The amplification was carried out in a thermal cycler (ABI 2720) with 5-min denaturation at 94 °C followed by forty-three cycles each consisting of 30 s denaturation at 94 °C, 30 s of annealing at 54 °C, and 30 seconds extension at 72 °C. Final extension was carried out at 72°C for 10 min.

**Fig. 2:**
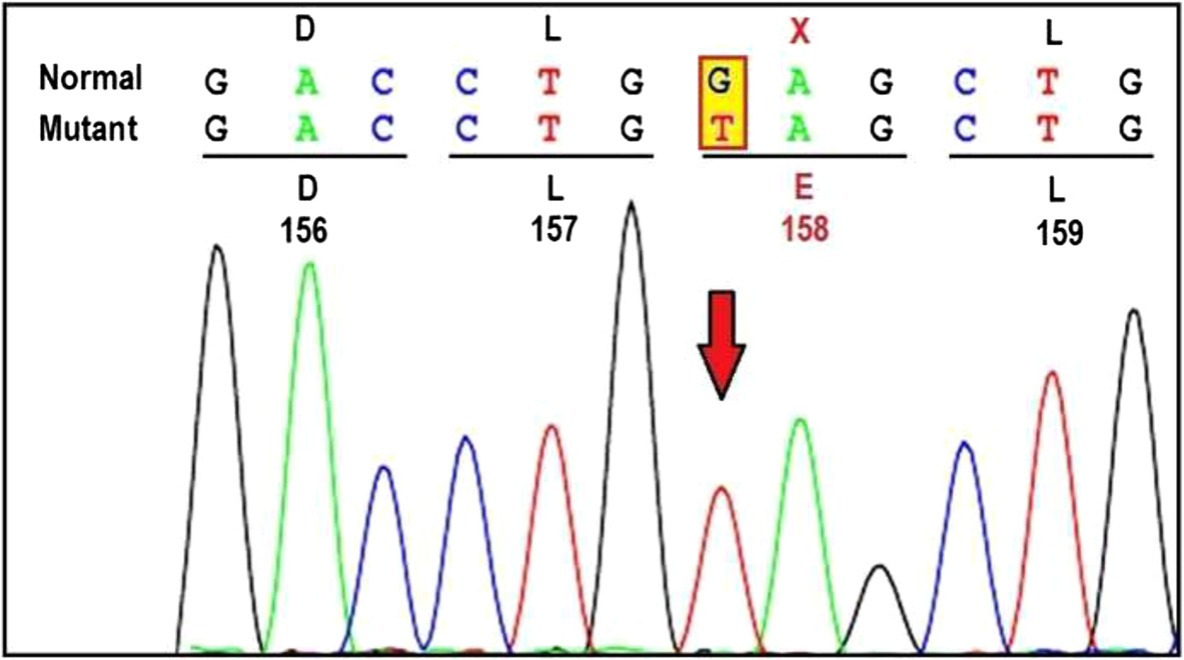
Bi-directional sequence chromatogram detected homozygous nonsense mutation viz. c.472 G > T (p.E158X) in exon-4 of *GM2A* gene

The *GM2A* gene analysis revealed a novel homozygous c.472G > T (p.E158X) nonsense mutation in exon-4confirming the diagnosis of GM2AP deficiency leading toGM2 gangliosidosis, AB variant (Fig. 2). Parents were found to be heterozygous for the same mutation. The deleterious effect of the mutation was further confirmed using bioinformatics tool; Mutation taster program.

## Discussion

GM2 activator deficiency as a cause of GM2 gangliosidosis is a rarely diagnosed sphingolipid disorder. Conzelmann and Sandhoff (1978) were the first one to present an evidence of defective activating factor necessary for degradation of GM2 ganglioside [10]. This was followed by demonstration of GM2 activator protein deficiency in liver cells in Non-Jewish child from England by Hechtmen et al. in 1982 [11]. Subsequently nine molecularly proven cases of GM2 activator deficiency from different parts of the world as summarized in Table 1 have been reported. Here we present tenth case having a novel mutation in *GM2A* gene. Clinically, proband was indistinguishable from classical infantile Tay-Sachs disease. However, due to the normal activity of Hex-A and total-hex enzymes in leucocytes, the c.DNA analysis of *GM2A* gene was carried out. The case under study showed homoallelic nonsense mutation (p.E158X) in *GM2A* gene, due to replacement of nucleotide Guanine (G) by Thymine (T) at c.DNA position 472 (c.472G > T). This is a novel pathogenic variant found to be responsible for a GM2AP deficiency in our patient with AB variant of GM2 gangliosidosis.

To the best of our knowledge, this mutation (p.E158X) is neither reported in any ethnic group, nor present in the dbSNP database (http://www.ncbi.nlm.nih.gov/SNP/). This variation has not been reported in the 1000 genome database and this region is conserved across species. *In-silico* analysis using the Mutation taster program suggests a probably damaging nature of the mutation. A subunit lacking the last 36 C-terminal residues is likely to make the protein non-functional. This premature stop codon is likely to have produced a truncated protein as the stop codon occurred near 3’end of the gene, which leads to unstable mRNAs. It is also likely that the proteins synthesized in the endothelium reticulum (ER) undergo a quality control check by the resident ER system that recognizes abnormal proteins and degrades them [12]. Though functional study has not been carried out in our case it is likely that nonsense point mutation in the present case seems to have produced a premature stop codon thus affecting the stability of the mutated protein or its Hex-A binding capabilities [13] or perhaps leading to early degradation in the ER or Golgi bodies [14], thus portraying the clinical presentation.

Only seven mutations in nine patients have been documented in the *GM2A* gene till date and all patients were found to be homozygous for individual mutant alleles associated with complete absence of GM2 ganglioside cleavage [2], [5, 6, 9, 14–19]. Earlier published reports have shown steady state levels of activator mRNA but none were found to have detectable activator cross reacting material (CRM) in patient’s cells except in a study reported by Chen et al. (1999) [9] where they have shown absence of detectable steady state mRNA nor any CRM.

The homoallelic nonsense mutation detected in the child was confirmed by the heterozygous state of both parents. This could be one of the reasons for the early death of the elder sister. Based on this, family was provided genetic counseling about the future recurrence risks of the disease and prevention by prenatal diagnosis.

## Conclusion

It can be concluded from this case that children with global developmental delay and unmyelinated white matter with normal hexosaminidase study need to be further investigated for GM2 activator protein deficiency.

## Abbreviations

CRM, Cross reacting material; ER, Endothelium reticulum; GM2AP, GM2 activator protein; Hex-A, β-hexosaminidase-A; Hex-T, β-hexosaminidase-total; mRNA, messenger RNA; MUG, 4-methylumbelliferyl-Nacetyl-β-D-glucosamine; MUGS, 4-methylumbelliferyl-N-acetyl-β-D-glucosamine-6-sulphate
